# Alcoholic liver disease and bilateral multifocal central serous retinopathy: a case report

**DOI:** 10.1186/1752-1947-7-43

**Published:** 2013-02-13

**Authors:** Despoina Gkotsi, Manish Gupta, Gerassimos Lascaratos, Andreas Syrogiannis, Baljean Dhillon

**Affiliations:** 1 Institute of Ophthalmology, 11-43 Bath Street, London, EC1V 9EL, UK; 2 NHS Greater Glasgow and Clyde, Stobhill and Gartnavel Hospital, 1053 Great Western Road, Glasgow, G12 0YN, UK; 3 Princess Alexandra Eye Pavilion, Edinburgh, EH3 9HA, UK

**Keywords:** Alcoholic liver disease, Ascites, Central serous retinopathy

## Abstract

**Introduction:**

We present a unique case of a patient with bilateral, multifocal central serous retinopathy in a patient with alcoholic liver disease.

**Case presentation:**

A 58-year-old Caucasian man with alcoholic liver disease, liver cirrhosis and ascites presented to the eye clinic. The ophthalmoscopic examination of both eyes revealed a symmetrical pattern of variably sized, slightly yellowish, translucent, raised lesions throughout the fundi which were confirmed to be caused by multifocal central serous retinopathy after optical coherence tomography and autofluoresence tests.

**Conclusion:**

This case highlights the possible link between central serous retinopathy and end-stage liver disease, with potential implications for the pathogenesis of central serous retinopathy in these patients.

## Introduction

Central serous retinopathy (CSR) is an exudative chorioretinopathy characterized by an exudative neurosensory retinal detachment with or without an associated detachment of the retinal pigment epithelium (RPE). It typically occurs in young, healthy adults and is usually idiopathic. The age range at the time of first diagnosis is generally from 22 to 83 years, and patients older than 50 years of age tend to have bilateral disease, systemic hypertension and a history of corticosteroid use [1]. Rare variants of CSR with chronic, bilateral, extrafoveal, multifocal and bullous retinal detachments have also been observed in patients undergoing cardiac transplantation [2]. Liver disease may be involved in sight-threatening eye diseases. The ophthalmic pathologies of cirrhosis in the literature include xerophthalmia, vitamin A deficiency and color blindness [3]. Abe *et al*. found retinopathy with hemorrhages and exudates in 31.8% of patients with hepatitis C, irrespective of liver cirrhosis [4]. According to Onder *et al*., retinopathy can be present not only in hepatitis C-positive patients but also in patients with other causes of liver cirrhosis, and soft exudates may develop in cirrhotic patients, probably due to loss of the synthetic function of the liver and the hemodynamic effects of portal hypertension [3]. Haimovici *et al*. showed a statistically significant relationship between alcohol intake and CSR [5]. Experimental studies have shown serous retinal detachment secondary to alteration of choroidal vascular permeability [6]. One of the studies suggests that ischemia at the level of the choroid can cause capillary and venous congestion with increased fluid transudation [7]. We report a unique case of bilateral multifocal CSR secondary to alcoholic chronic liver disease in a 58-year-old man.

## Case presentation

A 58-year-old Caucasian man was referred to the eye clinic in view of multiple raised yellowish lesions in both fundi. He had originally visited his optician for occasional flashes and floaters. He had recently been diagnosed with diet controlled type 2 diabetes mellitus and was on a low dose of amlodipine (5mg/day) for well controlled hypertension. His other drug history included analgesics (paracetamol, dihydrocodeine) and omeprazole. He admitted to heavy alcohol consumption in the past and had chronic liver disease with ascites.

His examination revealed that he had hepatomegaly with a palpable liver edge three fingerbreadths below the right costal margin, but no splenomegaly. An ultrasound of the liver showed generally increased echogenicity suggestive of liver cirrhosis. A computed tomography (CT) scan confirmed the presence of liver cirrhosis and showed evidence of esophageal varices, in keeping with decompensated chronic liver disease.

There was no evidence of a localized lesion in the liver, ruling out the possibility of both hepatocellular carcinoma and metastatic disease as causes of decompensation. His liver function tests (LFTs), including alkaline phosphatase (ALP), alanine aminotransferase (ALT) and γ-glutamyl transferase (GGT), had been elevated for several years. Interestingly, he was also found to have a marginally elevated plasma viscosity of 1.81mPa/s (normal range 1.5 to 1.72mPa/s) with no evidence of paraprotein.

**Figure 1:**
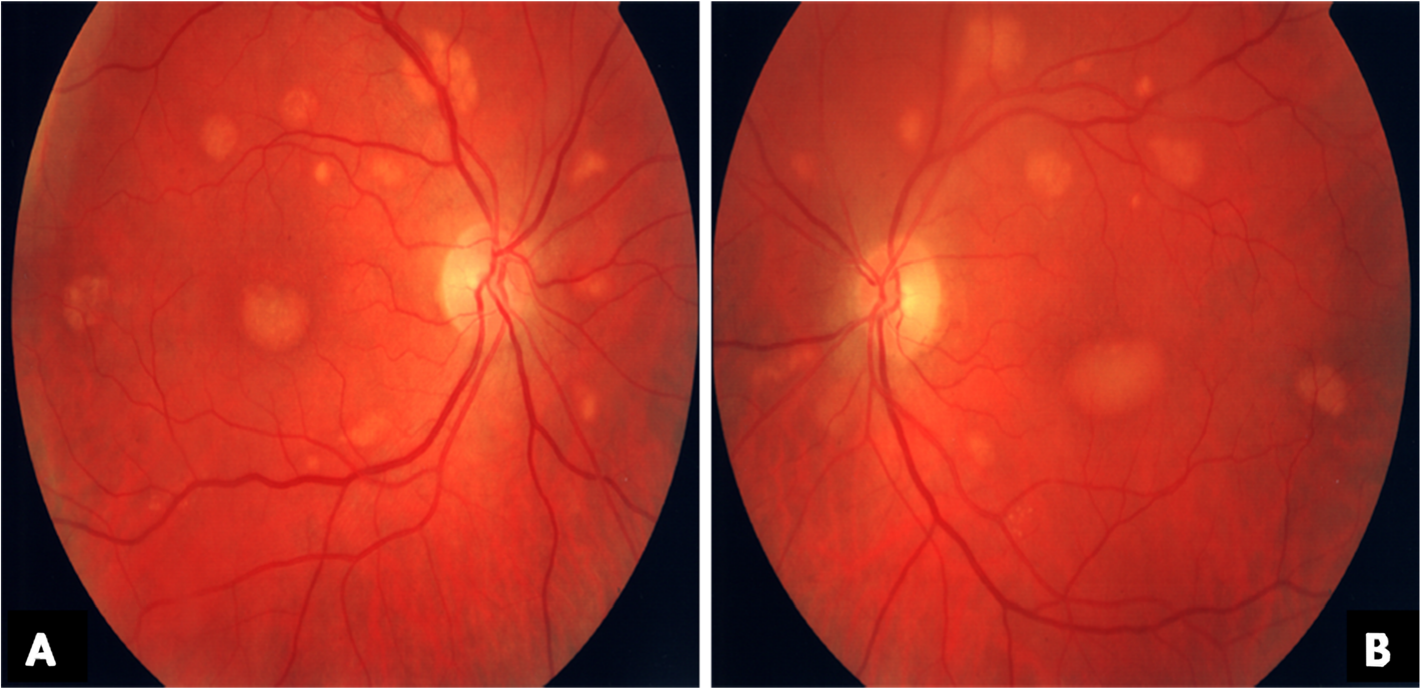
**(A) and (B) Fundal images.** Variably sized, slightly yellowish, translucent raised lesions throughout the fundi in both the right eye (**A**) and the left eye (**B**). Images were obtained at presentation.

**Figure 2:**
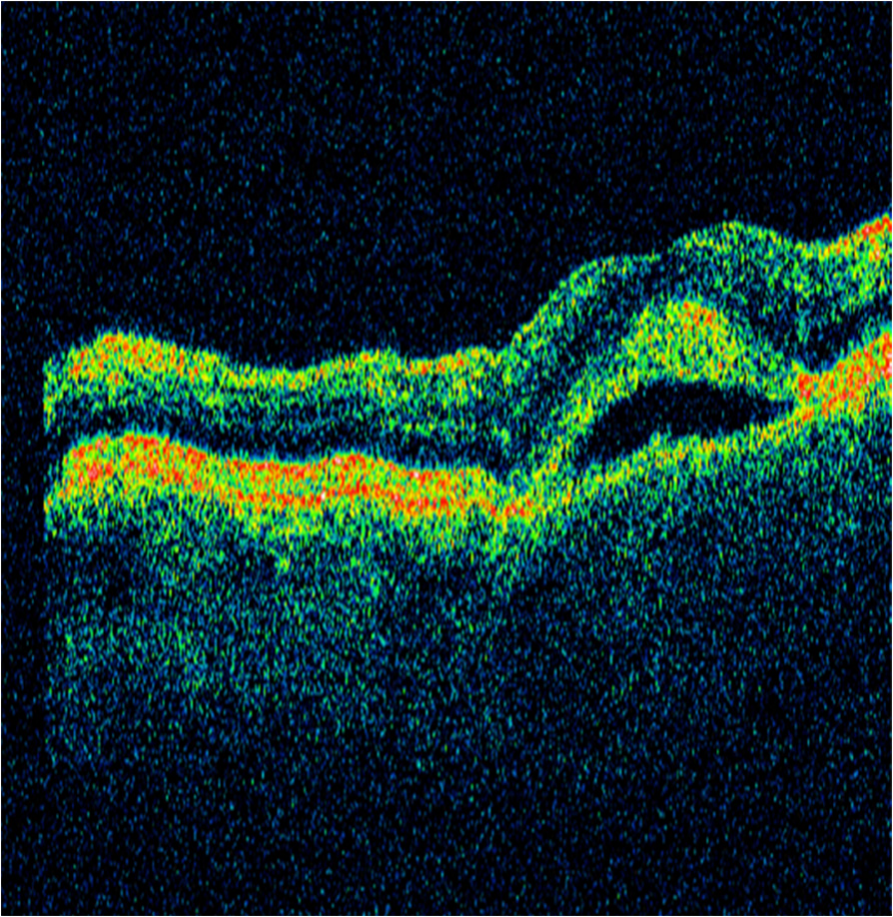
**Optical coherence tomography image.** Optical coherence tomography performed through the posterior pole demonstrated serous sensory detachment without any suggestion of retinal pigment epithelial detachment or retinal thinning. The image was obtained at presentation.

**Figure 3:**
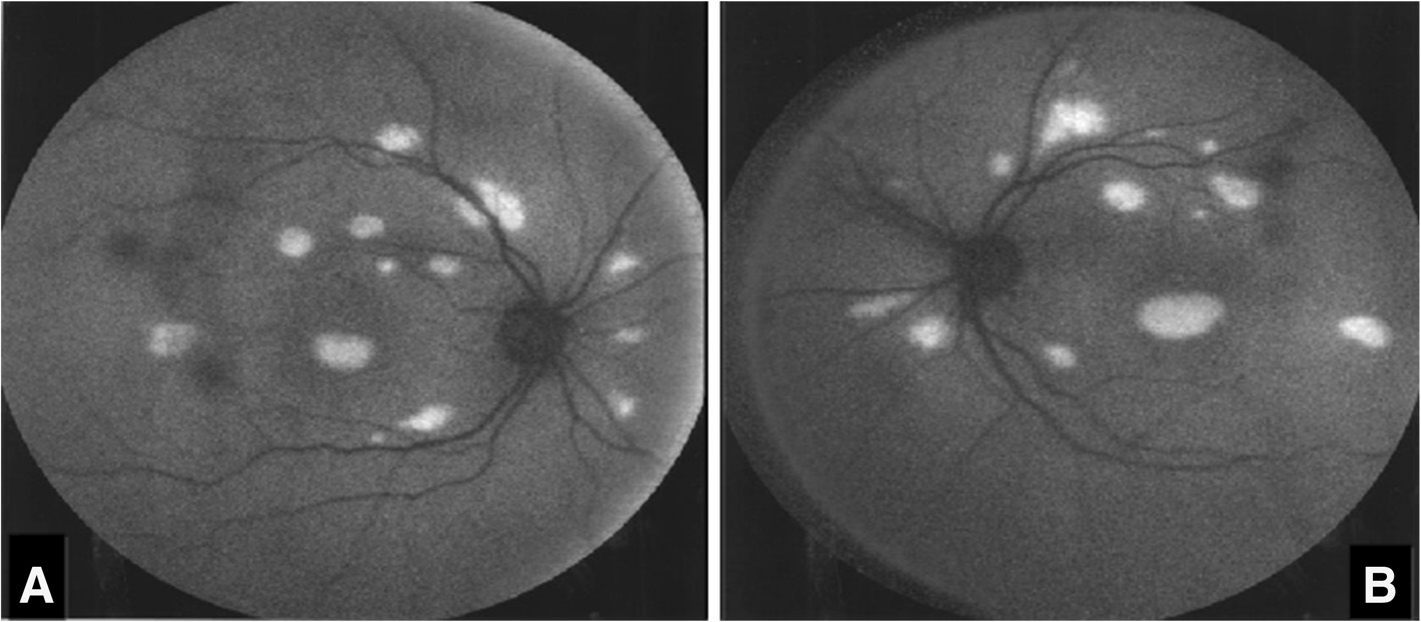
**(A) and (B) Fundus autofluorescence images.** Fundus autofluorescence recorded using a confocal scanning laser ophthalmoscope showed autofluorescence, which corresponds with the detached sensory retina in the right eye (**A**) and the left eye (**B**). The images were obtained at presentation.

His ocular examination was within normal limits for the anterior segment. His visual acuity was 6/6 in both eyes. Ophthalmoscopic examination of both eyes revealed a symmetrical pattern of dozens of variably sized, slightly yellowish, translucent raised lesions throughout the fundi (Figures 1A and 1B). These lesions were confirmed as multiple neurosensory retinal detachments on optical coherence tomography (OCT) (Figure 2) and fundus autofluorescence (Figures 3A and 3B). The patient was followed-up in the eye clinic and was asymptomatic until his last follow-up. Visual acuity, fundus and OCT findings were unchanged. As the visual acuity was good and there was no evidence of choroidal neovascularization, conservative management was recommended.

## Discussion

From a pathophysiological aspect, we hypothesize that in our patient the damaged liver produced less blood protein. This may have disturbed the body’s fluid balance, leading to alteration of choroidal vascular permeability, increased fluid transudation, serous fluid accumulation in the neurosensory retina and thus multifocal CSR [6]. Ammonia dysmetabolism has also been noted in patients with liver cirrhosis. It is perhaps interesting to note that patients with minimal hepatic encephalopathy, despite their presenting with normal mental and neurological status upon clinical examination, have been found to demonstrate inflammation and raised levels of ammonia in the blood caused by diminished clearance by the liver [8]. The increased serum levels of inflammatory markers (such as C-reactive protein, white blood cell count and IL-6) found in patients with liver cirrhosis [8] have been implicated in the breakdown of the blood–brain barrier. IL-6 and TNF-α are known to enhance fluid-phase permeability of isolated brain endothelial cells *in vitro*[9], suggesting that these and other inflammatory markers could also potentially contribute to changes in the outer blood–retina barrier and to an increase in choroidal vascular permeability, leading to CSR. Moreover, alcohol has been shown to be associated with nitric oxide–related abnormalities of choroidal blood flow autoregulation [10], thus providing an additional mechanism for the change in choroidal vascular permeability and the associated fluid leakage in the sub-RPE space and CSR development. Oxidative stress has also been implicated in liver cirrhosis [11]. Enhanced production of reactive oxygen species is thought to be involved in the nitration of tyrosine residues in intracellular proteins, thus affecting transastrocytic substrate transport and selective degradation of the permeability of the blood–brain barrier and potentially the outer blood–retina barrier [12,13].

## Conclusion

To the best of our knowledge, this is the first case of multifocal CSR related to alcoholic liver disease to be reported in the literature and has potential implications for the pathogenesis of CSR in these patients. Our patient had no other risk factors for CSR [5], such as systemic steroid, antihistamine or antibiotic use; history of autoimmune disease; untreated hypertension; or tobacco use. The differential diagnoses of acute exudative polymorphous paraneoplastic vitelliform maculopathy [14] and acute exudative polymorphous vitelliform maculopathy [15] could not be excluded in the absence of fluorescein angiography and electroretinography, although the non-progressive nature of the lesions during follow-up in our patient, the absence of subretinal yellowish deposits gravitating as a meniscus below the macula, and the normal visual acuity were not supportive of these diagnoses.

## Consent

Written informed consent was obtained from the patient for publication of this case report and any accompanying images. A copy of the written consent is available for review by the Editor-in-Chief of this journal.

## Abbreviations

ALP: Alkaline phosphatase; ALT: Alanine aminotransferase; CSR: Central serous retinopathy; CT: Computed tomography; GGT: γ-glutamyl transferase; IL: Interleukin; LFT: Liver function test; OCT: Optical coherence tomography; RPE: Retinal pigment epithelium; TNF: Tumor necrosis factor.

## Competing interests

The authors declare that they have no competing interests.

## Authors’ contributions

DG conceived and wrote the manuscript. MG wrote and reviewed the manuscript and provided final approval of the manuscript for publication. GL reviewed the manuscript and collected the references with final approval. AS followed up the patient and reviewed the manuscript. BD suggested changes and gave final approval of the manuscript for publication. All authors read and approved the final manuscript.
